# Supplementary material for: Metastatic Breast Cancer Recurrence after Bone Fractures
Source: Cancers (Basel). 2022 Jan 25;14(3):601. doi: 10.3390/cancers14030601 (PMC8833729; doi:10.3390/cancers14030601)
Supplement: Supplementary file 1 [file cancers-14-00601-s001.zip › cancers-1526871-supplementary.pdf]

Supplementary Materials

Table S1. International Statistical Classification of Diseases and Related Health Problems 10th Revision (ICD-10)-WHO.

**C00-C97 Malignant Neoplasms**
C00-C75 Malignant neoplasms, stated or presumed to be primary, of specified sites, except of lymphoid, haematopoietic and related tissue
C00-C14 Malignant neoplasms of lip, oral cavity and pharynx
C15-C26 Malignant neoplasms of digestive organs
C30-C39 Malignant neoplasms of respiratory and intrathoracic organs
C40-C41 Malignant neoplasms of bone and articular cartilage
C43-C44 Melanoma and other malignant neoplasms of skin
C45-C49 Malignant neoplasms of mesothelial and soft tissue
C50-C50 Malignant neoplasm of breast
C51-C58 Malignant neoplasms of female genital organs
C60-C63 Malignant neoplasms of male genital organs
C64-C68 Malignant neoplasms of urinary tract
C69-C72 Malignant neoplasms of eye, brain and other parts of central nervous system
C73-C75 Malignant neoplasms of thyroid and other endocrine glands
C76-C80 Malignant neoplasms of ill-defined, secondary and unspecified sites
C81-C96 Malignant neoplasms, stated or presumed to be primary, of lymphoid, haematopoietic and related tissue
C97-C97 Malignant neoplasms of independent (primary) multiple sites
**M80-M82 Disorders of bone density**
M80 Osteoporosis with pathological fracture
M81 Osteoporosis without pathological fracture
M82 Osteoporosis in diseases classified elsewhere

Table S2. Frequencies of types of fractures after breast cancer diagnosis.

	**Fracture at/After Diagnosis (ICD-10 S, T and M80) [a]** **N = 13,579**
**Type of first fracture (ICD-10 codes S or T or M80)**	
Extremities (lower & upper)	6778 (49.9)
Pathological fractures M80	2488 (18.3)
Spine/pelvis	1855 (13.7)
Rib/episternum	828 (6.1)
Hip/femoral neck	714 (5.3)
Head/Face	439 (3.2)
Not otherwise specified	394 (2.9)
**Number of different fractures**	
One specific ICD-code	11028 (81.2)
More than one specific ICD-code	2551 (18.8)

[a] Presented are N (percentage).

Table S3. Sensitivity analyses of associations between fractures and metastasis by excluding breast cancer diagnoses in 2015 and extended waiting period to metastasis of more than one year, and of patients who received endocrine therapy as an indicator for ER positive breast cancer.*.

		**Overall Metastasis**	**Lymph Node Metastasis**	**Distant Non-Bone Metastasis**	**Distant Bone Metastasis**
**Model 4: Extended waiting period >1 year, BCa diagnosis in 2015 included**	N (events)	78,238 (5,332)	78,238 (2,255)	78,238 (3,847)	78,238 (1,716)
**Fractures at/after BCa diagnosis**	HR (95%-CI)	**1.09 (1.02, 1.19)**	1.04 (0.92, 1.18)	**1.13 (1.03, 1.24)**	1.13 (0.99, 1.29)
**Model 5: Extended waiting period >1 year, BCa diagnosis in 2015 excluded**	N (events)	17,068 (593)	17,068 (293)	17,068 (375)	17,068 (169)
**Fractures at/after BCa diagnosis**	HR (95%-CI)	0.99 (0.71, 1.36)	0.86 (0.53, 1.39)	1.26 (0.88, 1.82)	1.18 (0.70, 2.00)
**Patients with endocrine therapy**					
**Model 6: Inclusion of BCa diagnosis in 2015**	N (events)	38,451 (4,341)	38,451 (2,008)	38,451 (2,938)	38,451 (1,604)
**Fractures at/after BCa diagnosis**	HR (95%-CI)	**1.15 (1.04, 1.26)**	1.05 (0.91, 1.22)	**1.24 (1.11, 1.38)**	**1.20 (1.04, 1.39)**
**Model 7: BCa diagnosis in 2015 excluded**	N (events)	12,934 (709)	12,934 (403)	12,934 (354)	12,934 (165)
**Fractures at/after BCa diagnosis)**	HR (95%-CI)	0.90 (0.63, 1.30)	0.66 (0.38, 1.16)	1.22 (0.80, 1.87)	**1.78 (1.07, 2.95)**

*All models were stratified by year of diagnosis (4 or 3 strata) and adjusted for age, osteoporosis, endocrine therapy, aromatase inhibitor, second tumors, other tumors prior diagnoses, and source of BCa diagnosis. Most covariates were included as time-dependent variables except of age, source of BCa diagnosis, and other tumors before diagnosis. Reference category is "No fracture", respectively. Bold: statistically significant exposure estimates.
